# Comparison of parameters for left ventricular volumes and function between echocardiography and cardiovascular magnetic resonance in a large group of cardiac patients

**DOI:** 10.1186/1532-429X-15-S1-E74

**Published:** 2013-01-30

**Authors:** Florian Andre, Cihan Celik, Hassan Abdel-Aty, Maria Fernanda Braggion Santos, Evangelos Giannitsis, Hugo A Katus, Henning Steen

**Affiliations:** 1 Department of Cardiology, University of Heidelberg, Heidelberg, Germany; 2 School of Medicine of Ribeirao Preto University of Sao Paulo, Sao Paulo, Brazil

## Background

Left ventricular (LV) volumes and function are of paramount importance for the correct diagnosis finding, cardiovascular risk stratification and assessment of future cardiac events as well as for therapeutic guidance. In clinical routine, echocardiography (EC) is the method of choice due to its wide availability and its straight-forward examination. However, cardiovascular magnetic resonance (CMR) is the gold-standard for the evaluation of cardiac volumes and function. In this study we compared the LV volumes and functional parameters obtained by EC with CMR results in a large group of cardiac patients.

## Methods

We included 1017 subjects (736 male, 281 female) retrospectively receiving both EC and CMR who presented to our cardiology department. EC was performed on four different systems (Philips IE 33, GE Vivid 7, GE Vivid I, GE Vivid S5) and images were analyzed by experienced readers. LV end-diastolic, end-systolic, and stroke volumes (EDV, ESV, SV) as well as ejection fraction (EF) were measured by two different EC techniques, a) a modified Simpson's rule and b) the Teichholz formula, and compared to the CMR results. CMR imaging was performed on a 1.5 T whole-body MRI using a standard SSFP sequence and short-axis views were obtained for the LV quantification. Analyses of variances were performed between groups and a p<0.05 was regarded as statistically significant. Furthermore a Bland-Altman analysis was conducted plotting against CMR as the gold-standard.

## Results

EC and CMR volumetric and functional parameters of 973 patients were compared whereas study times were 1.45 (0-9) days apart. CMR-EDV, -ESV and -SV were significantly higher (p<0.01) when compared to EC. The EF differed significantly between all three techniques (p<0.02) with higher values for CMR. Interestingly, Teichholz-EDV, -ESV and -SV were significantly closer to the CMR results than the Simpson measurements. The Bland-Altman plots showed a relation between the accordance of the parameters measured by CMR and EC and their absolute value with the best agreement for medium values (see Figure).

**Figure 1:**
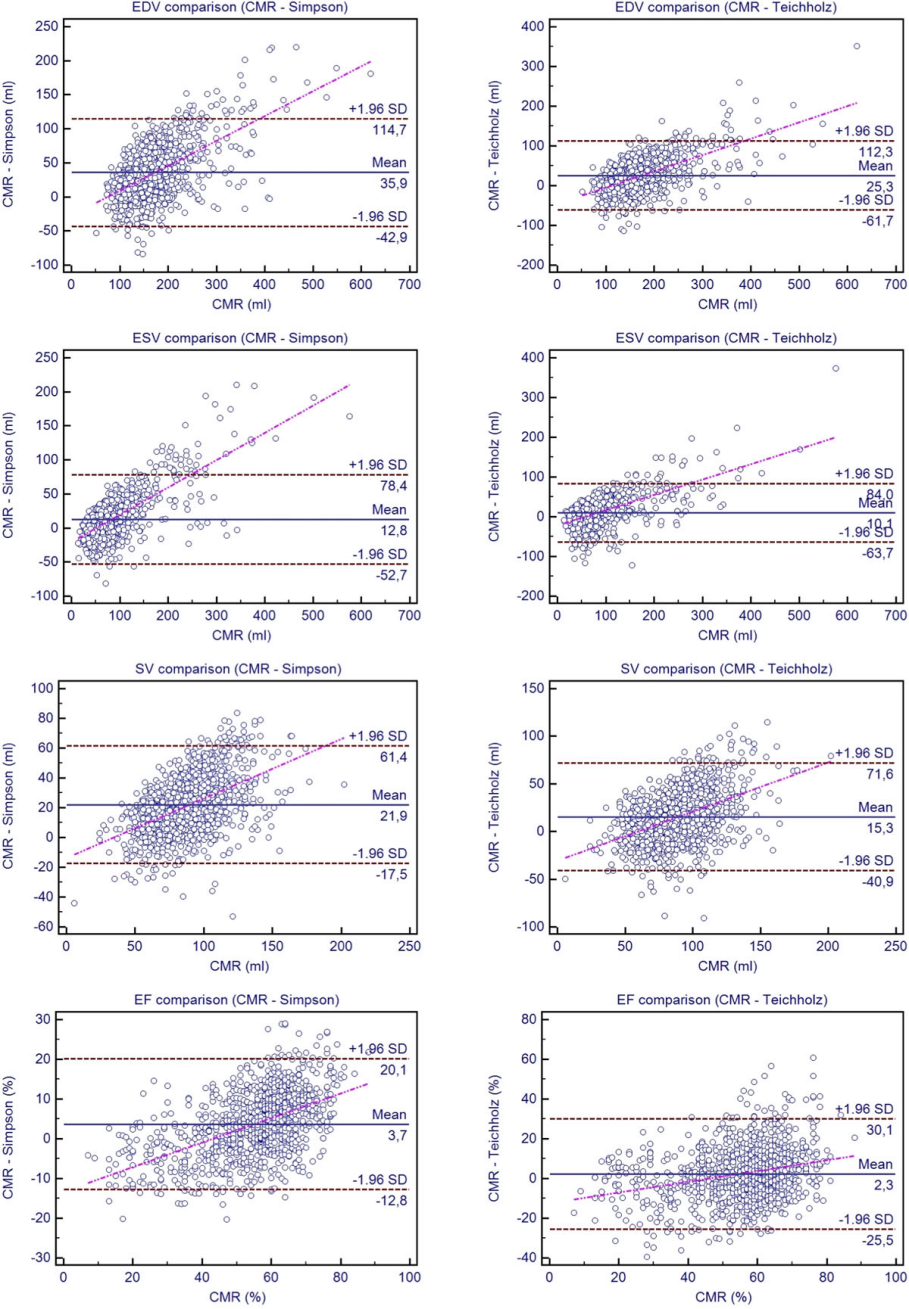
Bland-Altman-plot of LV volumetric and functional parameters measured by EC (Simpson and Teichholz formula) against CMR as reference method

## Conclusions

CMR provides higher values than EC for LV volumes and ejections fraction presumably due to its superior blood tissue contrast. Furthermore the values obtained by different EC formulas were significantly different. Interestingly, the discrepancy between CMR and EC was dependent of the absolute LV volume and functional values showing a greater deviation for lower and higher values. As an exact measurement especially in these clinical scenarios is of great therapeutic importance (e.g. indication for an ICD) further studies are needed.

## Funding

none

**Table 1:** Comparison LV volumetric and functional parameters acquired by CMR and EC (Simpson and Teichholz formula)

n=1017	CMR	Simpson	Teichholz
EDV (ml)	172.9±63.6	136.9±52.0	147.8±51.9
ESV (ml)	83.7±59.8	70.8±42.7	73.8±47.3
SV (ml)	89.0±24.6	67.1±22.9	73.7±28.4
EF (%)	54.8±14.0	51.1±12.1	52.4±17.1

All values are shown as mean±standard deviation.

